# Supplementary material for: Host-mediated selection impacts the diversity of Plasmodium falciparum antigens within infections
Source: Nat Commun. 2018 Apr 11;9:1381. doi: 10.1038/s41467-018-03807-7 (PMC5895824; doi:10.1038/s41467-018-03807-7)
Supplement: Supplementary file 1 — Supplementary Information [file 41467_2018_3807_MOESM1_ESM.pdf]

Supplementary Material

Early *et al.* Host-mediated selection impacts the diversity of *Plasmodium falciparum* antigens within infections.

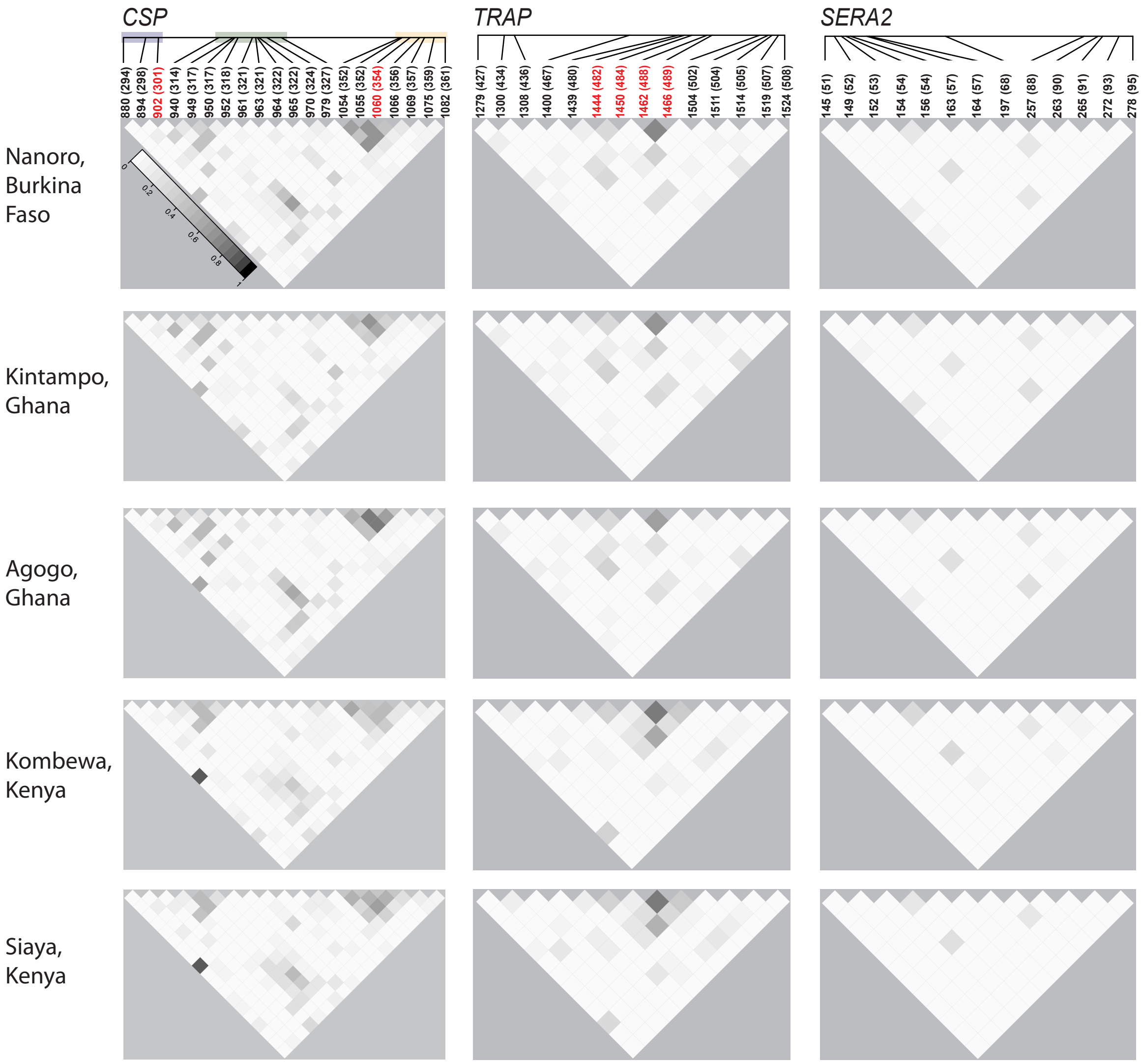

Supplementary Figure 1. LD plots for nucleotides within the *CSP*, *TRAP*, and *SERA2* amplicon regions for each of the five study sites. LD between polymorphic nucleotides was calculated as Q*, an extension of r^2 that allows for multiple alleles per site. Corresponding amino acid positions are in parentheses. Positions marked in red showed evidence for altered intra-host diversity at the amino acid level. For *CSP*, shading marks the positions within the DV10 (blue), Th2R (green), and Th3R (yellow) epitope regions. Only nucleotide positions with a major allele frequency <0.98 were included in the analysis.

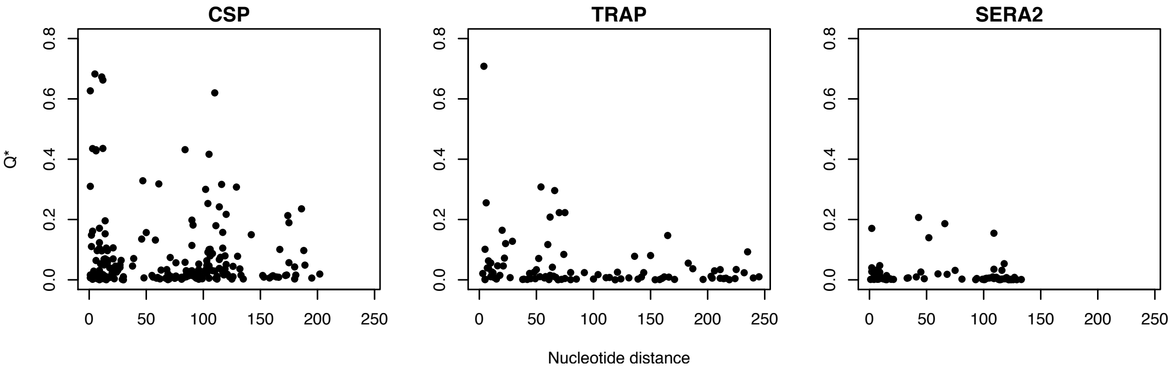

Supplementary Figure 2. Plots of LD (Q*) decay with nucleotide distance for the *CSP*, *TRAP*, and *SERA2* amplicon regions. LD between polymorphic nucleotides was calculated as Q*, an extension of r^2 that allows for multiple alleles per site.

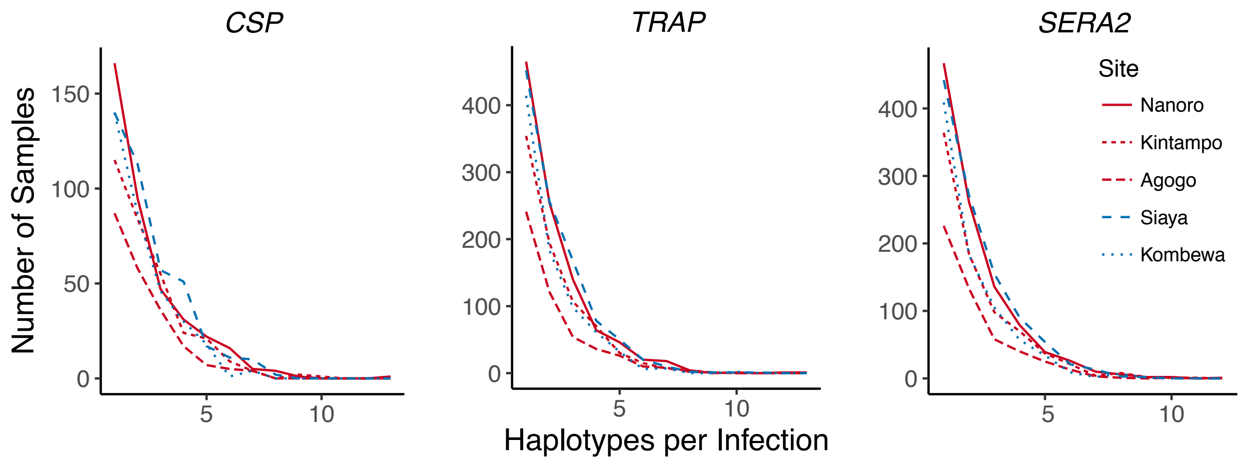

Supplementary Figure 3. Number of haplotypes sequenced per infection for each amplicon region.
