# Supplementary material for: Host-mediated selection impacts the diversity of Plasmodium falciparum antigens within infections
Source: Nat Commun. 2018 Apr 11;9:1381. doi: 10.1038/s41467-018-03807-7 (PMC5895824; doi:10.1038/s41467-018-03807-7)
Supplement: Supplementary file 3 — Description of Additional Supplementary Files [file 41467_2018_3807_MOESM3_ESM.pdf]

Description of Additional Supplementary Files

File Name: Supplementary Data 1
Description: **Nucleotide diversity and population differentiation for variants in the *CSP* amplicon.** Population genetic statistics for each of the nucleotide variants identified in CSP using amplicon sequencing.

File Name: Supplementary Data 2
Description: **Nucleotide diversity and population differentiation for variants in the *TRAP* amplicon.** Population genetic statistics for each of the nucleotide variants identified in TRAP using amplicon sequencing.

File Name: Supplementary Data 3
Description: **Nucleotide diversity and population differentiation for variants in the *SERA2* amplicon**. Population genetic statistics for each of the nucleotide variants identified in SERA2 using amplicon sequencing.

File Name: Supplementary Data 4
Description**: Number of unique haplotypes containing the nucleotide pairs used in the LD analysis.** For each combination of two variant positions, this table gives the number of times the pair appeared on a distinct haplotypic background. These numbers complement the LD analysis reported in the main text by showing that the LD patterns are not due to the expansion of a single haplotype.

File Name: Supplementary Data 5
Description: **List of Pf3k samples used in the genome-wide analysis.** These sample IDs passed quality filtering, showed evidence of containing only a single clonal lineage, and were used for the genome-wide population genetic analysis.

File Name: Supplementary Data 6
Description: **Unique *CSP, TRAP*, and *SERA2* haplotypes found in samples collected from children enrolled in the control arm of the RTS,S/AS01 phase 3 vaccine trial.** This table provides the sequence information for the haplotypes from control vaccinated individuals that were used in the amplicon analysis.

File Name: Supplementary Data 7
Description: **Haplotype IDs and select associated metadata for samples collected from children enrolled in the control arm of the RTS,S/AS01 phase 3 vaccine trial.** This list provides metadata for the control samples used in the analysis including haplotype ID, haplotype count, patient age, patient sex, and study location.
